# A Case of Autologous Cultivated Limbal Stem Cell Transplantation

**Published:** 2010-04

**Authors:** Ahmad Kheirkhah, Farid Karimian

**Affiliations:** Assistant Professor, Farabi Eye Hospital, Tehran University of Medical Sciences, Tehran, Iran; Associate Professor, Ophthalmic Research Center, Shahid Beheshti University of Medical Sciences, Tehran, Iran

## CASE PRESENTATION

**Figure 1:**
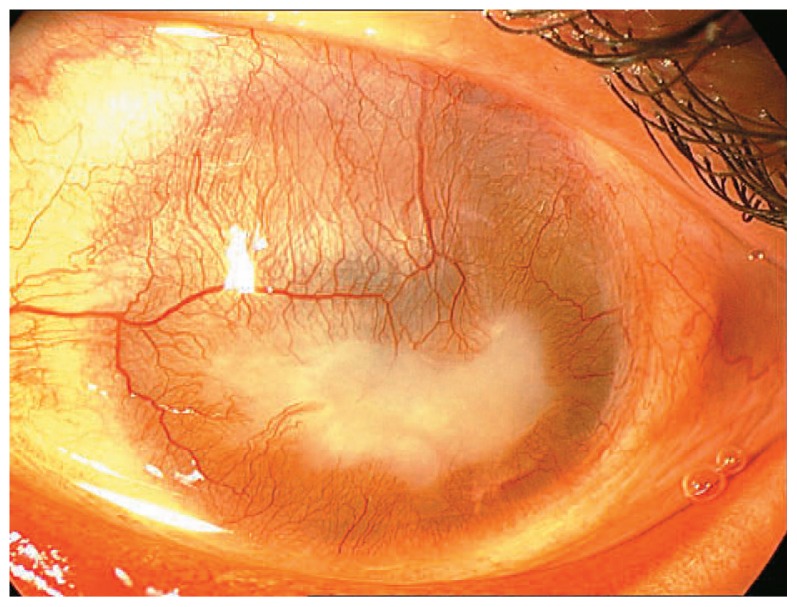
Slitlamp appearance of the left cornea demonstrating total vascularized opacity.

The patient presented herein, is a 29-year-old man with history of unilateral alkaline chemical injury 14 years ago. Upon referral, visual acuity was 20/20 and the examination was unremarkable in the right eye. Vision was hand motions (HM) in his left eye and a small angle exotropia (10–15 Prism Diopters) was evident. Slitlamp examination revealed severe corneal opacification and vascularization (Fig. 1). Intraocular pressure (IOP) was 13 and 10 mmHg in the right and left eyes, respectively. B-scan ultrasonography ruled out gross posterior segment pathologies and impression cytology revealed total limbal stem cell deficiency (LSCD) in the left eye.

**Figure 2:**
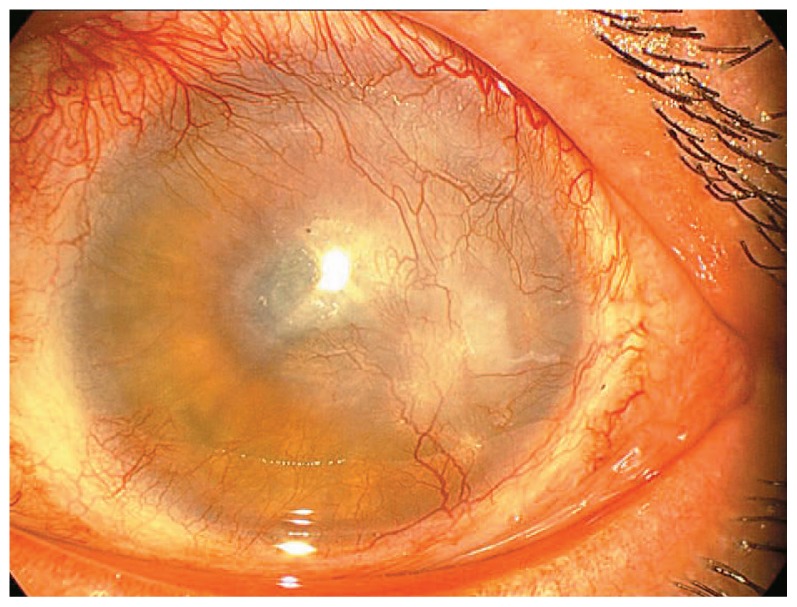
Three months after stem cell transplantation epithelial transparency has increased, while corneal vascularization and opacification have decreased.

The injured eye underwent cultivated autologous stem cell transplantation on amniotic membrane (AM) after punctal cauterization. The patient received a topical steroid (betamethasone 0.1%, Sina-Daru, Tehran, Iran), and an antibiotic (chloramphenicol 0.5%, Sina-Daru, Tehran, Iran). Antibiotic drops were continued for 1 to 2 weeks until complete epithelialization. Steroid drops were tapered off according to the level of ocular inflammation over 6–8 weeks. A preservative-free artificial tear (Artelac, Bausch and Lomb, USA) was used for lubrication as needed. The patient received systemic prednisolone 1 mg/kg (Sina-Daru, Tehran, Iran) for 3 to 4 weeks. Visual acuity in the left eye improved to counting fingers at 75 cm along with modest decrease in corneal vascularization and opacity 3 months after the procedure (Fig. 2).

Due to deep corneal stromal opacification, penetrating keratoplasty (PKP) was performed 6 months later. One month after PKP, visual acuity improved to 20/120. The patient complained of diplopia which was due to improvement in vision and the small angle exotropia. Six weeks later, IOP increased to 32 mmHg which was controlled by topical timolol 0.5% (Sina-Daru, Tehran, Iran) two times per day and systemic acetazolamide 250mg, three times per day. Cup-disc ratio was 0.3. Systemic acetazolamide was discontinued due to elevated liver enzymes and replaced by topical dorzolamide 2% (Sina-Daru, Tehran, Iran) two times per day.

**Figure 3:**
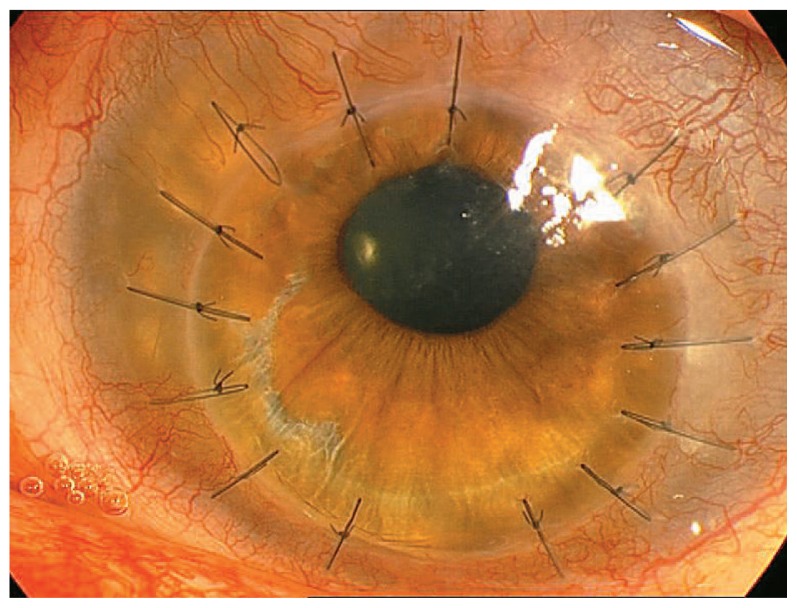
Slitlamp appearance 5 months after corneal transplantation.

Eight weeks after corneal transplantation, the patient developed epithelial rejection, which was treated with topical steroids. Four months after surgery, IOP rose to 30 mmHg and brimonidine 0.2% (Alphagan, Allergan Inc., Irvine, USA) and latanoprost 0.005% (Xalatan, Pfizer Inc., New York, USA) were added to the regimen. Five months after PKP, visual acuity improved to 20/80 but IOP was poorly controlled (Fig. 3). The patient underwent Ahmed Glaucoma Valve (AGV) surgery. Two months later, a mild posterior subcapsular cataract was observed. Five month after AGV, corneal endothelial and subepithelial rejection were observed which was aggressively treated with systemic prednisolone and topical betamethasone. Overall, the patient experienced three attacks of corneal endothelial rejection 5, 9 and 16 months after AGV. There was a slightly progressive vascularization in the graft periphery 8 months after PKP.

Considering multiple episodes of corneal endothelial rejection and progressive corneal conjunctivalization in an eye with defective ocular surface, systemic cyclosporine (Sandimmune^®^, Novartis Pharma Stein AG, Stein, Switzerland) 300 mg per day was started. One month later, it was replaced by mycophenolate mofetil (Cellcept^®^, Hoffmann-La Roche Inc., Nutley, USA) 2 grams per day due to elevated liver enzymes. Two weeks later liver enzymes levels improved but 2 months thereafter, rose critically again and mycophenolate was also discontinued. After that, the patient was put on fluorometholone 0.25% eye drops once daily together with topical cyclosporine 2%.

**Figure 4:**
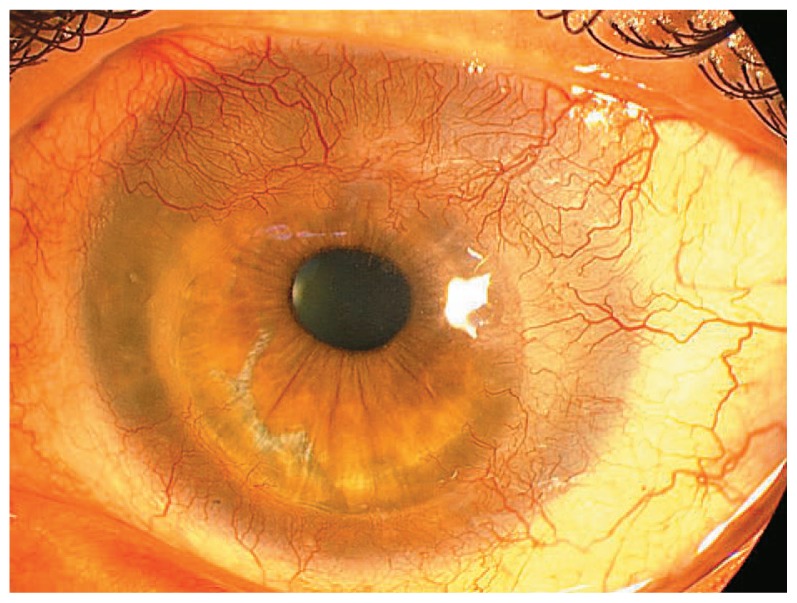
The left cornea at last visit demonstrates peripheral conjunctivalization and a clear center.

One year after PKP, impression cytology confirmed corneal conjunctivalization in the superior and inferior temporal quadrants by the presence of goblet cells on the corneal side of the specimens. About 20 months after PKP, vascularization and conjunctivalization was continuing but did not reach the central 5 mm zone of the cornea (Fig. 4). At final visit, visual acuity was maintained at 20/80 and IOP was 16 mmHg without any glaucoma medications.

Herein we present the views of two anterior segment specialists on the management of LSCD in this patient.

### Ahmad Kheirkhah, MD

To maximize the outcomes of transplantation of *ex vivo* cultured limbal epithelial cells (LECs), several issues need to be considered. First of all, the ocular surface should be optimized before performing any kind of limbal stem cell transplantation. Although both puncti of the recipient eye were cauterized prior to transplantation, it is unclear whether the ocular surface was actually ready for surgery. For example, it has not been mentioned whether the recipient eye had any other factor which may interfere with ocular surface health, such as surface inflammation, symblepharon, closure and blinking problems, or lid margin abnormalities.

After any form of *ex vivo* culture of LECs and before transplantation of the product to the ocular surface, it is necessary to demonstrate that the product really contains viable stem cells. A number of methods have been employed for this purpose such as functional assay of colony forming ability of transplanted cells, immunohistological assays, evidence of donor cell survival, and confocal scanning. In this case report, the evaluation method for the product has not been stated. Transplantation of a product with inadequate viable stem cells can result in long-term failure, which finally developed in this patient.

Although the surgical technique for transplantation of *ex vivo* cultured LECs is fairly similar in different studies, postoperative medical regimens may differ among them. The basic principles of this regimen include immediate control of inflammation, prophylaxis against infection, and mechanical protection of the graft. Application of autologous serum eye drops may further optimize the environment for stem cell survival. The early postoperative course of the patient has not been mentioned which may be a clue to final graft survival.

The patient in this report showed improvement in visual acuity with moderate decrease in corneal vascularization and opacification 3 months after surgery (Fig. 2). However, these could not be considered to be absolute measures of success. There were still many areas of corneal vascularization with a clinical appearance of residual conjunctivalization (Fig. 2). There is no data on fluorescein staining patterns or the results of impression cytology at this postoperative time point. Due to lack of these data, there is a possibility that the patient was still suffering from residual LSCD at that time. It should be mentioned that in addition to clinical findings, a more definitive way to evaluate the success of surgery is to perform impression cytology on the corneal surface postoperatively. This test, which was not performed in the case report, is necessary before proceeding with corneal transplantation. This may explain why the patient finally developed partial LSCD.

Performing an eccentric full-thickness graft in a vascularized cornea (Fig. 3) should be considered as a high risk corneal graft. This is why the patient developed multiple episodes of graft rejection. In these cases, if there is no full-thickness corneal involvement, it is always preferable to perform lamellar rather than full-thickness corneal transplantation.

The postoperative management of this immunologically high risk graft is vital in determining the outcome. The main measure to be used for prevention of rejection in high risk grafts is immunosuppression. Although corticosteroids have traditionally been used for this purpose, new agents have been available in the past years. In high risk patients, topical steroids are used frequently in the postoperative period, followed by their long-term use if there is no contraindication. Sometimes, low dose steroids need to be used indefinitely after surgery. Therefore, potential complications of steroids, such as cataracts, glaucoma, delayed wound healing, and infectious keratitis should seriously be considered. Systemic steroids can also be used as an adjunct to topical therapy, particularly in high risk cases with concomitant systemic inflammatory diseases. Recently, steroids with lower rates of complications have been introduced; examples include loteprednol etabonate and fluorometholone which can be used for keratoplasty patients who require long-term maintenance therapy.

In addition to corticosteroids, cyclosporine A (CsA), a calcineurin inhibitor, has been used extensively in organ transplantations including high risk corneal allografts. Many studies have demonstrated the effectiveness of CsA, either topically or systemically, to reduce rejection rates in high risk keratoplasty patients. Although 0.05% CsA is commercially available, a 2% concentration has been shown to be effective for this purpose. Oral CsA may be used in very high-risk cases when topical medications may not be sufficient to prevent corneal graft rejection. However, oral CsA is associated with a number of dose-related side effects, most commonly nephrotoxicity, hypertension, and hepatotoxicity, which developed in this case. Various other systemic immunosuppressive agents have been used in patients with high risk corneal grafts such as tacrolimus, azathioprine, mycophenolate mofetil, and rapamycin, sometimes in combination. However, all of these agents may be associated with adverse effects, which developed in this case after using mycophenolate. In my personal experience, the preferable initial immunosuppressive medication is topical 2% CsA which may be used alone or in combination with topical steroids. This may be why the patient did not develop any episode of graft rejection while on topical CsA.

This case report shows how difficult it may be to manage patients with significant ocular surface disease. A comprehensive multidisciplinary approach is necessary to deal with these grave eye conditions.

### Farid Karimian, MD

This 29-year-old man with severe vascularization, conjunctivalization and diffuse haziness of the cornea has unilateral total LSCD. He had history of chemical injury 14 years before presentation. At this stage, any type of corneal transplantation (penetrating or lamellar) would have a short survival and may fail rapidly. Because the right eye is normal in appearance and has no history of exposure to chemical agents, limbal stem cells could be harvested for *ex vivo* expansion. This technique which has become available in some centers in recent years, enables patients to make benefit of their own stem cells. This technique brings hope for traditionally poor chance cases with LSCD. At this stage it is very important to optimize the status of the fornices. Severe symblepharon and foreshortening of the fornices prevents adequate tear film lubrication and lid function. Therefore, reconstruction of the fornices and release of symblepharon using amniotic membrane can be helpful.

After limbal stem cell transplantation, it would be better to use preservative-free medications. The medications used in this case are not preservative-free and can be toxic to the transplanted tissue.

After PKP in cases who are considered to be high risk due to previous limbal stem cell (LSC) transplantation, immunosuppressive treatment must be used. Steroids alone are not enough and can be used in the immediate post-PKP period, but systemic or topical immunosuppression with medications such as cyclosporine A (Sandimmune^®^) or mycophenolate mofetil (Cellcept^®^) are important preventive measures against rejection of corneal transplants. It would be better to start these medications earlier and prior to immune system activation.

IOP rise with coticosteroids can be due to steroid responsiveness. This risk is higher with topical betamethasone than other steroids. If the use of these types of medications is mandatory, alternative medications, e.g. fluorometholone or loteprednol can be considered.

In summary, the management of LSCD has remarkably changed over the recent years. Cultivation of LSC and application of immunosuppressive medications will help clinicians improve the final outcomes. Glaucoma is a sight-threatening complication in these cases. Manipulation of cicatrized and scarified conjunctiva, extensive use of steroids, and multiple operations are important factors predisposing to glaucoma.

### Editor’s Note

Surgical management of LSCD depends on the laterality and severity of corneal involvement. Partial LSCD can be managed using amniotic membrane transplantation (AMT) or sequential epitheliectomy. In partial or total unilateral cases, conjunctival-limbal autograft (CLAU) is a good choice. Recently, transplantation of limbal epithelial stem cells cultivated on carriers such as AM or transplantation of *ex vivo* cultured autologous oral mucosal epithelial cells has been considered as alternative procedures for unilateral or bilateral LSCD.

The main objective of any kind of stem cell transplantation is to supply new corneal epithelium for a prolonged, if not indefinite, period of time so that patients can be relieved from annoying photophobia and regain useful vision. Cultivation of a small part of the limbus may provide epithelial progenitor cells, which might survive for a while on the ocular surface. In eyes with superficial corneal vascularization and pannus, a single procedure is frequently enough. However, if there is concomitant deep corneal stromal scarring, penetrating or lamellar keratoplasty is needed to restore vision. Drawback to CLAU and conjunctival-limbal allograft (CLAL) is that due to removal of fairly large segments of limbal tissue, the donor eye is at risk of surgically induced LSCD. The use of autologous cultivated limbal stem cell transplantation has been suggested to overcome this limitation. Cultivating a small amount of limbal tissue provides adequate limbal stem cells for treatment of total LSCD.

Use of AM as a carrier medium for cultivated limbal stem cells entails the following advantages. Its stromal matrix is similar to conjunctival basement membrane and is a suitable medium for growth of limbal stem cells, it is antigen-free and resorbed gradually. AM facilitates ocular surface epithelialization, it down-regulates inflammatory cytokines, facilitates epithelial cell differentiation and may be used for ocular surface reconstruction.

The use of *ex vivo* cultured limbal epithelial stem cells for treatment of corneal LSCD in humans was first described by Pellegrini et al in 1997. Since then several additional reports on the use of this technology to treat patients have been published. In addition, other studies have reported the transplantation of *ex vivo* cultured autologous oral mucosal epithelial cells to treat LSCD. Numerous reviews have dealt with the scientific theory and evidence behind this treatment. In almost all studies, the authors conclude that this method is a successful procedure to treat unilateral corneal stem cell deficiency. Some key questions still need to be answered. The exact proportion of stem cells present in *ex vivo* cultured limbal epithelial cell sheets is unclear and needs to be determined. The behavior of limbal epithelial stem cells post-transplantation also needs to be elucidated. It has been proposed that the success of this treatment relies on the re-integration of exogenous cultured limbal stem cells into the ocular surface, and that these cells function to continuously replenish the corneal epithelium. It is interesting that despite the different methodologies employed, the success rate and outcomes are remarkably similar.

The surgeon subsequently performed optical PKP in this case. He selected fresh donor cornea with a very good coverage of epithelial cells; therefore some transient amplifying cells (TACs), which support corneal clarity have been theoretically transferred by the corneal graft. It is not possible to judge whether the cause of corneal clarity is related to TACs transferred by the corneal graft or due to transplanted epithelial progenitor cells cultivated on the AM. However, it seems that the period of corneal clarity was relatively longer than usual corneal grafts without stem cell transplantation.

In summary, cultivated stem cell transplantation on AM might be an effective way to improve the health of the injured ocular surface. Despite uncertainties, published clinical evidence supports the continued advancement of this procedure for treatment of corneal epithelial stem cell deficiency. To date, this procedure has been regarded as experimental, but in the light of the clinical outcomes, it seems that it has the potential to become a viable option for treatment of many patients with severe limbal stem cell deficiency.
